# Secondary Metabolites from a Marine-Derived Endophytic Fungus *Penicillium chrysogenum* QEN-24S

**DOI:** 10.3390/md9010059

**Published:** 2010-12-27

**Authors:** Shu-Shan Gao, Xiao-Ming Li, Feng-Yu Du, Chun-Shun Li, Peter Proksch, Bin-Gui Wang

**Affiliations:** 1 Key Laboratory of Experimental Marine Biology, Institute of Oceanology, Chinese Academy of Sciences, Nanhai Road 7, Qingdao 266071, China; E-Mails: xisea01@126.com (S.-S.G.); lixmqd@yahoo.com.cn (X.-M.L.); fooddfy@126.com (F.-Y.D.); lichunshun@ms.qdio.ac.cn (C.-S.L.); 2 Graduate School of the Chinese Academy of Sciences, Yuquan Road 19A, Beijing 100049, China; 3 Institute for Pharmaceutical Biology and Biotechnology, Heinrich-Heine-University Duesseldorf, Universitaetsstreet 1, 40225 Duesseldorf, Germany; E-Mail: proksch@uni-duesseldorf.de

**Keywords:** marine endophyte, *Penicillium chrysogenum*, secondary metabolites

## Abstract

*Penicillium chrysogenum* QEN-24S, an endophytic fungus isolated from an unidentified marine red algal species of the genus *Laurencia*, displayed inhibitory activity against the growth of pathogen *Alternaria brassicae* in dual culture test. Chemical investigation of this fungal strain resulted in the isolation of four new (**1**–**3** and **5**) and one known (**4**) secondary metabolites. Their structures were identified as two polyketide derivatives penicitides A and B (**1** and **2**), two glycerol derivatives 2-(2,4-dihydroxy-6-methylbenzoyl)-glycerol (**3**) and 1-(2,4-dihydroxy-6-methylbenzoyl)- glycerol (**4**), and one monoterpene derivative penicimonoterpene (**5**). Penicitides A and B (**1** and **2**) feature a unique 10-hydroxy- or 7,10-dihydroxy-5,7-dimethylundecyl moiety substituting at C-5 of the α-tetrahydropyrone ring, which is not reported previously among natural products. Compound **5** displayed potent activity against the pathogen *A. brassicae*, while compound **1** exhibited moderate cytotoxic activity against the human hepatocellular liver carcinoma cell line.

## 1. Introduction

**Figure 1:**
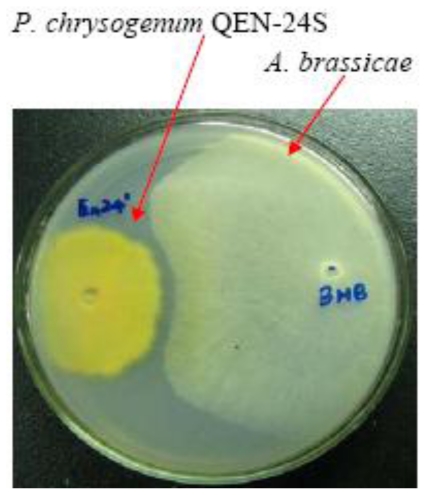
*P. chrysogenum* QEN-24S inhibitory activity against growth of the pathogen fungus *A. brassicae*.

Marine derived fungi have been proven to be a rich source of structurally unique and biologically active secondary metabolites [1]. In the past years, a number of new metabolites have been isolated and identified and their biological activities have been evaluated [2–4]. As part of our recently initiated program to assess the chemical and biological diversity of endophytic fungi derived from marine algae [5–12], *Penicillium chrysogenum* QEN-24S, an endophytic fungus obtained from the inner tissue of an unidentified marine red algal species of the genus *Laurencia*, attracted our attention. This fungus displayed inhibitory activity against the growth of pathogenic fungus *Alternaria brassicae* in our initial dual culture test (Figure 1). This phenomenon prompted us to examine the chemical metabolites of this fungal strain. The fungus *P. chrysogenum* was previously reported as the source of several β-lactam antibiotics, most significantly penicillin [13,14]. Besides the antibacterial activity, it was also reported that *P. chrysogenum* served as a source of bioactive substances with other activities such as antifungal, anti-HIV, and cytotoxic activity [15,16].

**Figure 2:**
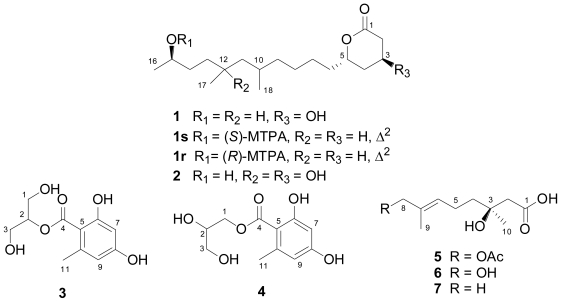
Structures of the isolated compounds **1**–**5** and the reference compounds **6** and **7**.

We wish to report herein the isolation and structure determination of four new compounds, namely, penicitides A and B (**1** and **2**), 2-(2,4-dihydroxy-6-methylbenzoyl)-glycerol (**3**), and penicimonoterpene (**5**) (Figure 2), from the rice culture fermentation of *P. chrysogenum* QEN-24S. In addition, a related known compound 1-(2,4-dihydroxy-6-methylbenzoyl)-glycerol (**4**) [17] was also isolated and identified. The structures of these compounds were established on the basis of extensive spectroscopic analysis. The absolute configuration at C-15 of penicitide A (**1**) was determined by application of the modified Mosher’s method. The inhibitory activity of these metabolites against two pathogens *A. brassicae* and *Aspergullus niger* was determined. In addition, the cytotoxic activity against seven tumor cell lines was also evaluated.

## 2. Results and Discussion

### 2.1. Bioassay-Guided Isolation

The endophyte *P. chrysogenum* QEN-24S displayed obvious activity against the pathogen fungus *A. brassicae* in our initial dual culture test (Figure 1). This endophyte was therefore submitted to a large scale fermentation for bioactive compounds isolation. The EtOAc extract derived from the rice culture of the fungal strain was suspended in MeOH-H_2_O (9:1, v/v) and was extracted with *n*-hexane to remove the non-polar fraction. The MeOH-soluble fraction displayed moderated inhibitory activity against the pathogenic fungus *A. brassicae*. This fraction was then subjected to various separation procedures by column chromatography on silica gel, Lobar LiChroprep RP-18, and Sephadex LH-20, to afford four new (**1**–**3**, and **5**) and one known secondary metabolites (**4**), as shown in Figure 2. By detailed analysis of the spectroscopic data, the structure for the known compound **4** was determined as 1-(2,4-dihydroxy-6-methylbenzoyl)-glycerol (**4**) [17].

### 2.2. Structural Elucidation of the New Compounds

**Figure 3:**
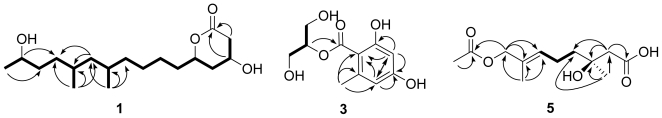
Key ^1^H-^1^H COSY (bold lines) and HMBC (arrows) correlations of compounds **1**, **3** and **5**.

Compounds **1** and **2** were obtained as colorless oils. The IR spectrum of **1** showed absorption bands for OH (3405 cm^−1^) and C=O (1716 cm^−1^) functionalities in the molecules. The low-resolution ESI-MS displayed ion peaks at *m*/*z* 337 [M + Na]^+^ and 651 [2M + Na]^+^. Its molecular formula was determined as C_18_H_34_O_4_ on the basis of positive HR-ESI-MS, indicating two degrees of unsaturation. The ^13^C-NMR (Table 1) along with the DEPT experiments revealed the presence of 18 carbon atoms including one carbonyl carbon, five *sp*^3^ methines, nine *sp*^3^ methylenes, and three methyl groups. The remaining one unsaturation demonstrated that **1** is a monocyclic compound. The ^1^H-^1^H COSY correlations revealed the presence of a contiguous sequence of the proton signals comprising from H-2 to H-16, as shown in Figure 3. Further ^1^H-^1^H COSY correlations from H_3_-17 to H-12 and from H_3_-18 to H-10 unambiguously demonstrated two methyl groups H_3_-17 and H_3_-18 attached to C-12 and C-10, respectively. The HMBC correlations from H-2 and H-3 to C-1 established the linkage of C-1 to C-2 and C-3. Finally, the chemical shifts of C-5 (δ_C_ 75.68, d) and C-1 (δ_C_ 170.33, s) established the connection of C-1 and C-5 via an ester linkage to form a tetrahydropyran-2-one moiety as shown in Figure 3.

**Figure 4:**
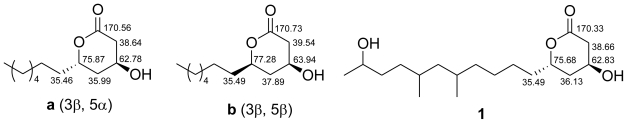
Comparison of ^13^C-NMR chemical shifts (in CDCl_3_) of **1** with two analogues (**a** and **b**) to establish the relative configurations at C-3 and C-5 in **1**.

The relative configuration of the two chiral centers C-3 and C-5 in the tetrahydropyran ring was proposed by comparison of its ^13^C-NMR data with those published for its analogues [18] sharing common partial structures from C-1 to C-8 (Figure 4). The relative configurations of C-3 and C-5 were deduced as 3β and 5α, respectively, as shown in Figure 2, according to the corresponding chemical shifts as shown in Figure 4. This was supported by the fact that no NOE correlations could be observed between H-3 and H-5 in the NOESY experiment. However, the relative configuration for C-10, C-12, and C-15 in the side chain remains unknown. Based on the above evidence, the structure for compound **1** was established as 4β-hydroxy-6α-(10-hydroxy-5,7-dimethylundecyl)tetrahydropyran-2-one, which was named as penicitide A, as shown in Figure 2.

**Figure 5:**
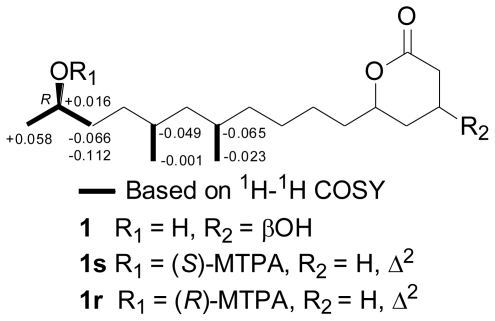
Values of Δδ_H(_*_S_*_–_*_R_*_)_ (measured in CDCl_3_) of the MTPA esters (α-methoxy-α-(trifluoro-methyl)phenylacetate) of compound **1**.

The absolute configuration at C-15 of penicitide A (**1**) was determined to be *R* by application of modified Mosher’s method, as shown in Figure 5. However, due to the dehydration between H-2 and OH-3 during the acylation, attempts to determine the absolute configurations for C-3 and C-5 failed.

Compound **2** was assigned the molecular formula C_18_H_34_O_5_, having one oxygen unit more than **1**, on the basis of positive HR-ESI-MS experiments. Detailed comparison of the ^1^H- and ^13^C-NMR data (Table 1) revealed that the structure of **2** was very similar to **1**. However, the methine carbon signal at δ_C_ 30.11 (C-12) in the ^13^C-NMR spectrum of **1** was replaced by an oxygenated quaternary carbon at δ_C_ 72.37 in **2**. Accordingly, the methine proton at δ_H_ 1.45 in **1** disappeared in the ^1^H-NMR spectrum of **2**. Moreover, the doublet signal at δ_H_ 0.85 (*J* = 6.4 Hz) for H_3_-17 in **1** was replaced by a downfield singlet at δ_H_ 1.15 in the ^1^H-NMR spectrum of **2**. The above evidences indicated that an OH substituent at C-12 in **2**. The HMBC correlations from H-11 and H_3_-17 to C-12 supported this conclusion. The configuration for the chiral centers at C-3 and C-5 was determined to be the same as that of **1** by NOESY experiment, as well as by detailed comparison of the NMR data with that of **1**. The structure for compound **2** was therefore established as 4β-hydroxy-6α-(7,10-dihydroxy-5,7- dimethylundecyl)-tetrahydropyran-2-one as shown in Figure 2, which was named penicitide B.

Penicitides A and B (**1** and **2**) bear a unique 10-hydroxy- or 7,10-dihydroxy-5,7-dimethylundecyl moiety substituting at C-5 of the α-tetrahydropyrone ring. This structure feature is not reported previously among natural products. These compounds appear to be α-tetrahydropyrone polyketides that are derived from a mixed-precursor biosynthesis including acetate and propionate building blocks [19]. Propionate polyketides are well known components from marine invertebrates and actinomycetes [20,21], but they have been rarely isolated from fungi as far as we know.

Compounds **3** and **4** were obtained as a colorless oily mixture. Attempts to separate the two compounds by different CC steps as well as by semi-preparative HPLC with different solvent systems failed. Fortunately, compound **3** could be distinguished from **4**, aided by 2D NMR experiments including ^1^H-^1^H COSY, HSQC, and HMBC, and by their different ratio (1:3) as indicated by the ^1^H- and ^13^C-NMR spectra. Most NMR signals are well-resolved. The structure of compound **4** was readily identified as 1-*O*-(2,4-dihydroxy-6-methylbenzoyl)-glycerol by detailed NMR spectral data analysis (Table 2) and comparison with our recent report [17]. Low-resolution ESI-MS displayed pseudo-molecular ion peaks at *m*/*z* 265 [M + Na]^+^ and 507 [2M + Na]^+^ for both compounds. The molecular formula was determined as C_11_H_14_O_6_ on the basis of positive HR-ESI-MS, suggesting five degrees of unsaturation. The structure of **3** was independently assigned by analysis of the ^1^H- and ^13^C-NMR data (Table 2) as well as by HSQC and HMBC correlations. The only difference between the two isomers was the connectivity of the glycerol moiety with the 2,4-dihydroxy-6-methylbenzoyl unit. The symmetric nature of the NMR data for the glycerol moiety (H-1/H-3 and C-1/C-3) in **3** as well as the observed ^3^*J* correlation from H-2 to C-4 in the HMBC spectrum (Figure 3) established the linkage of 2,4-dihydroxy-6-methylbenzoyl group to C-2 of the glycerol unit. Thus, the structure of compound **3** was assigned as 2-(2,4-dihydroxy-6-methylbenzoyl)-glycerol, as shown in Figure 2.

Compound **5** was obtained as colorless oil. Its IR spectrum showed absorption bands for OH (3178 cm^−1^) and C=O (1727 cm^−1^) functionalities in the molecules. The low-resolution ESI-MS displayed ion peaks at *m*/*z* 245 [M + H]^+^, 267 [M + Na]^+^, and 511 [2M + Na]^+^. Its molecular formula was determined as C_12_H_20_O_5_ on the basis of positive HR-ESI-MS. The planar structure was determined mainly by comparison with the literature reports of the known compounds (*E*)-3,8-dihydroxy-3,7- dimethyl-6-octenoic acid (**6**) [22] and (*S*,*E*)-3-hydroxy-3,7-dimethyl-6-octenoic acid (**7**) [23] as well as by the NOESY spectral data. Detailed comparison of the ^1^H-NMR data of **5** (Table 3) with that of **6** [22] revealed that these two structures were very similar, except an extra acetyl methyl signal at δ_H_ 2.07 was observed in the ^1^H-NMR spectrum of **5**. Further analysis of the ^13^C-NMR data of **5** indicated that an extra acetyl unit was attached to the monoterpene skeleton of **6**. The location of the acetyl unit was clearly determined at C-8 by the long range correlations from the acetoxyl methyl proton to C-8 in the HMBC spectrum. The observed NOE correlation from H-6 to H-8 in the NOESY spectrum revealed that the double bond was in *trans*-geometry. Finally, the absolute stereochemistry at C-3 was assigned to be *S* by comparison of the optical rotation of **5** ([α]_D_^20^ = +1.4, MeOH) with that of **7** ([α]_D_^25^ = +1.8, MeOH) [23].

Compound **5**, which was named penicimonoterpene, pertains to a class of naturally occurring monoterpene derivatives named citronellic acid formed by the oxidation of citronellal with a carboxyl group at C-1 and an olefinic double bond at C-6 as shown in Figure 2, which has been isolated from ascomycete [23] and fungus [24].

### 2.3. Biological Activities of the Isolated Compounds

The biological activity of the isolated compounds **1**–**5** was examined in antifungal and cytotoxicity bioassays. In the initial antifungal screening, penicimonoterpene (**5**) displayed potent activity against pathogen *A. brassicae* with an inhibition zone of 17 mm in diameter at the concentration of 20 μg/disk, while penicitide A (**1**) displayed moderate activity with an inhibition zone of 6 mm in diameter at the concentration of 20 μg/disk (Table 4). The cytotoxicity evaluation was also performed and the results indicated that penicitide A (**1**) possessed activity against HepG2 cell line with the IC_50_ of 32 μg/mL. The other compounds displayed no appreciable activity.

## 3. Experimental Section

### 3.1. General

Column chromatography was performed on commercial silica gel (Qingdao Haiyang Chemical Group Co., China; 200–300 mesh), Lobar LiChroprep RP-18 (40–63 μm; Merck), and Sephadex LH-20 (Pharmacia). Optical rotations were measured on an Optical Activity AA-55 polarimeter. UV Spectroscopic data were obtained on a Lengguang Gold Spectrumlab 54. IR spectra were performed on a JASCO FT/IR-4100 Fourier Transform infrared spectrometer. NMR spectra were acquired on a Bruker Avance-500 spectrometer at 500 MHz for ^1^H and 125 MHz for ^13^C. MS data were recorded on a VG Autospec-3000 mass spectrometer.

### 3.2. Fungal Material

The endophytic fungus *Penicillium chrysogenum* QEN-24S was isolated, by use of a standard procedure as in our previous report [9], from the inner tissue of an unidentified marine red algal species of the genus *Laurencia* collected from the Weizhou Island of southern China sea. Fungal identification was carried out by use of a molecular biological protocol by DNA amplification and sequencing of the ITS region as in our previous report [9]. The sequence data derived from the fungal strain has been submitted to and deposited at GenBank with accession number GU985086. A BLAST search result showed that the sequence was similar (99%) to the sequence of *P. chrysogenum* (compared to AY373903.1 GI: 34809383). The strain is preserved at the Key Laboratory of Experimental Marine Biology, Institute of Oceanology of the Chinese Academy of Sciences, with accession number QEN-24S.

Mass growth of the fungus for the isolation and identification of new metabolites was carried out in Erlenmeyer flasks (1 L each). The fungus was grown on rice solid medium (to 100 g commercially available rice, 0.6 g of peptone and 100 mL of distilled water was added, then kept overnight prior to autoclaving) at room temperature under static conditions for 30 days.

### 3.3. Extraction and Isolation

The rice culture (10 flasks) of the fungal strain *P. chrysogenum* QEN-24S was extracted with EtOAc. The crude extract obtained was dried and partitioned between *n*-hexane and 90% MeOH. The 90% MeOH-soluble material (2 g) was subjected to column chromatography (CC) over silica gel, eluting with different solvents of increasing polarity from petroleum ether (PE) to MeOH to yield 10 fractions (Frs. 1–10) on the basis of TLC analysis. Fr. 5 (0.3 g) was further purified by CC on silica gel eluting with a CHCl_3_-MeOH gradient (from 80:1 to 1:1), Sephadex LH-20 (MeOH), and Lobar LiChroprep RP-18 with a H_2_O-MeOH gradient (from 1:1 to 0:1) to afford **1** (10.5 mg) and a CC-unseparable mixture of **3** and **4** (11.1 mg). Fr. 8 (0.4 g) was further purified by CC on silica gel by eluting with a CHCl_3_-MeOH gradient (from 80:1 to 5:1), Sephadex LH-20 (MeOH), and preparative TLC (plate: 20 × 20 cm, developing solvents: CHCl_3_-MeOH, 10:1) to afford **2** (5.4 mg) and **5** (12.4 mg).

*Penicitide A* (**1**). Colorless oil; [α]_D_^20^: +42.9 (*c* 0.14, MeOH); IR (KBr) ν_max_ 3405, 2927, 2865, 1716, 1457, 1376, 1253, 1064, 937, 852, 725, 586 cm^−1; 1^H- and ^13^C-NMR data, see Table 1; ESI-MS *m*/*z* 337 [M + Na]^+^, 651 [2M + Na]^+^; HR-ESI-MS: *m*/*z* 337.2345 [M + Na]^+^ (calcd for C_18_H_34_O_4_Na^+^, 337.2354).

*Penicitide B* (**2**). Colorless oil; [α]_D_^20^: +100.0 (*c* 0.08, MeOH); IR (KBr) ν_max_ 3394, 3297, 2931, 2861, 1724, 1635, 1458, 1377, 1057, 802, 489 cm^−1; 1^H- and ^13^C-NMR data, see Table 1; ESI-MS *m*/*z* 353 [M + Na]^+^, 683 [2M + Na]^+^; HR-ESI-MS: *m*/*z* 353.2297 [M + Na]^+^ (calcd for C_18_H_34_O_5_Na^+^, 353.2303).

*2*-(*2*,*4*-*Dihydroxy*-*6*-*methylbenzoyl*)-*glycerol* (**3**). Colorless oil; UV λ_max_ (MeOH) nm (log ɛ): 214 (4.79), 264 (4.50); ^1^H- and ^13^C-NMR data, see Table 4; ESI-MS *m*/*z* 265 [M + Na]^+^, 507 [2M + Na]^+^; HR-ESI-MS: *m*/*z* 265.0689 [M + Na]^+^ (calcd for C_11_H_14_O_6_Na^+^, 265.0688).

*Penicimonoterpene* (**5**). Colorless oil; [*α*]_D_^20^: +1.4 (*c* 0.83, MeOH); IR (KBr) ν_max_ 3178, 2969, 2938, 1727, 1442, 1381, 1238, 1025, 949, 852 cm^−1; 1^H- and ^13^C-NMR data, see Table 5; ESI-MS *m*/*z* 245 [M + H]^+^, 267 [M + Na]^+^, 511 [2M + Na]^+^; HR-ESI-MS: *m*/*z* 267.1203 [M + Na]^+^ (calcd for C_12_H_20_O_5_Na^+^, 267.1208).

### 3.4. Preparation of the (*R*)- and (*S*)-MTPA Ester Derivatives of Compound **1** [*25*]

(*S*)-(+)-α-Methoxy-α-(trifluoromethyl)phenylacetyl chloride (10 μL) and 4-(dimethylamino)-pyridine (2 mg) were added to penicitide A (**1**, 1.1 mg) which was dissolved in dry pyridine (400 μL). The mixture was kept at room temperature for 12 h. The acylation product was purified by preparative TLC on silica gel [eluent: petroleum ether/EtOAC (4:1, v/v)] to yield corresponding (*R*)-Mosher ester **1r**. Treatment of **1** (1.2 mg) with (*R*)-MTPA-Cl (10 μL) as described above yielded the corresponding (*S*)-Mosher ester **1s**.

### 3.5. Dual Culture Test

Dual culture test of *P. chrysogenum* QEN-24S against *A. niger* and *A. brassicae* was carried out as literature report [26]. Pathogens and endophyte *P. chrysogenum* QEN-24S were inoculated at the periphery of the PDA-medium plate (200 g potato, 20 g dextrose, 20 g agar, and 1 L sea water), and then cultured at 27 °C for 5 days.

### 3.6. Antifungal Assay

Antifungal assay against *A. niger* and *A. brassicae* was carried out using the well diffusion method [27]. Amphotericin B (AMPB) was used as positive control.

### 3.7. Cytotoxicity Assay

The cytotoxic activities against NCI-H460 (human non-small cell lung cancer), SMMC-7721 (human hepatoma), SW1990 (human pancreatic cancer), DU145 (human prostate carcinoma), HepG2 (human hepatocellular liver carcinoma), Hela (human epithelial carcinoma), and MCF-7 (human breast adenocarcinoma) cell lines were determined according to previously reported methods [28].

## 4. Conclusions

In summary, four new (**1**–**3** and **5**) and one known (**4**) secondary metabolites were characterized from the algal-derived endophytic fungus *P. chrysogenum* QEN-24S and their chemical structures were solved by spectroscopic and chemical analysis. Among these, penicimonoterpene (**5**) were natural inhibitors of pathogenic fungus *A. brassicae*. Penicitide A (**1**) also displayed moderate activity against *A. brassicae*. All of these results are consistent with the initial dual culture test, in which *P. chrysogenum* QEN-24S displayed obvious inhibitory activity against the growth of pathogenic fungus *A. brassicae*. In addition, compound **1** exhibited moderate selective cytotoxic activity against HepG2 tumor cell line.

## Figures and Tables

**Table 1:** ^1^H- and ^13^C-NMR data of compounds **1** and **2** a.

No.	1 b		2 c	

	^1^H	^13^C	^1^H	^13^C
1		170.33 s		172.31 s
2	2.61 dd (17.6, 5.1)	38.66 t	2.54 dd (17.7, 3.5)	37.81 t
	2.73 dd (17.6, 3.7)		2.71 dd (17.7, 4.7)	
3	4.38 m	62.83 d	4.25 m	61.96 d
4	1.73 m	36.13 t	1.72 m	35.14 t
	1.95 m		1.93 m	
5	4.68 tdd (7.9, 5.2, 3.0)	75.68 d	4.69 m	76.46 d
6	1.59 m	35.49 t	1.61 m	35.32 t
	1.73 m		1.70 m	
7	1.41 m	25.11 t	1.36 m	24.86 t
8	1.30 m	26.39 t	1.37 m	26.49 t
9	1.08 m	36.55 t	1.19 m	38.58 t
	1.30 m		1.40 m	
10	1.54 m	29.88 d	1.66 m	28.37 d
11	0.93 m	44.79 t	1.30 m	48.71 t
	1.22 m		1.48 m	
12	1.45 m	30.11 d		72.37 s
13	1.14 m	32.67 t	1.45 m	38.19 t
	1.35 m		1.60 m	
14	1.38 m	36.55 t	1.47 m	33.19 t
	1.48 m		1.52 m	
15	3.76 m	68.56 d	3.69 m	67.85 d
16	1.19 d (6.9)	23.54 q	1.16 d (6.2)	22.17 q
17	0.85 d (6.4)	20.28 q	1.15 s	25.91 q
18	0.84 d (6.4)	20.26 q	0.97 d (6.6)	21.01 q

a Measured at 500 MHz for ^1^H and 125 MHz for ^13^C with reference to the solvent signals, δ in ppm and *J* in Hz;

b Measured in CDCl_3_;

c Measured in CD_3_OD.

**Table 2:** ^1^H- and ^13^C-NMR data of compounds **3** and **4** a.

No.	3		4	

	^1^H	^13^C	^1^H	^13^C
1	3.87 brs	61.67 t	4.35 dd (11.5, 6.0) 4.46 dd (11.5, 4.5)	67.38 t
2	5.20 m	78.01 d	4.03 m	70.73 d
3	3.87 brs	61.67 t	3.66 d (5.4)	64.28 t
4		172.06 s		172.43 s
5		106.06 s		105.68 s
6		166.16 s		166.29 s
7	6.23 d (2.5)	101.68 d	6.25 d (2.5)	101.73 d
8		163.26 s		163.35 s
9	6.28 d (2.5)	112.32 d	6.29 d (2.5)	112.37 d
10		144.68 s		144.93 s
11	2.51 s	24.36 q	2.51 s	24.45 q

a Measured in acetone-*d*_6_ at 500 MHz for ^1^H and 125 MHz for ^13^C with reference to the solvent signals, δ in ppm and *J* in Hz.

**Table 3:** ^1^H- and ^13^C-NMR data of compound **5** a.

No.	^1^H	^13^C	No.	^1^H	^13^C
1		176.37 s	7		130.56 s
2	2.57 d (15.8) 2.50 d (15.8)	44.72 t	8	4.44 s	70.09 t
3		71.07 s	9	1.66 s	13.83 q
4	1.61 m	41.12 t	10	1.29 s	26.53 q
5	2.14 m	22.27 t	OAc	2.07 s	20.92 q
6	5.44 t (6.9)	128.84 d			171.16 s

a Measured in CDCl_3_ at 500 MHz for ^1^H and 125 MHz for ^13^C with reference to the solvent signals, δ in ppm and *J* in Hz.

**Table 4:** Inhibitory activity of compounds **1**–**5** against the pathogenic fungi *A. niger* and *A. brassicae* a.

	AMPB b	1	2	3 + 4	5
*A. niger*	24	+	−	−	+
*A. brassicae*	18	6	−	−	17

a The diameter of the zone of inhibition is indicated in mm. To each disk, 20 μg of sample compound was loaded. The plus (+) means slight inhibition and the minus (−) means no inhibition;

b AMPB: amphotericin B was used as positive control.
